# Breath-Taking Perspectives and Preliminary Data toward Early Detection of Chronic Liver Diseases

**DOI:** 10.3390/biomedicines9111563

**Published:** 2021-10-28

**Authors:** Antonio Murgia, Yusuf Ahmed, Kelly Sweeney, Louise Nicholson-Scott, Kayleigh Arthur, Max Allsworth, Billy Boyle, Olga Gandelman, Agnieszka Smolinska, Giuseppe Ferrandino

**Affiliations:** 1 Owlstone Medical, 183 Cambridge Science Park, Milton Road, Cambridge CB4 0GJ, UK; antonio.murgia@owlstone.co.uk (A.M.); yusuf.ahmed@owlstone.co.uk (Y.A.); kelly.sweeney@owlstone.co.uk (K.S.); louise.nicholson@owlstone.co.uk (L.N.-S.); kayleigh.arthur@owlstone.co.uk (K.A.); max.allsworth@owlstone.co.uk (M.A.); billy.boyle@owlstone.co.uk (B.B.); olga.gandelman@owlstone.co.uk (O.G.); agnieszka.smolinska@owlstone.co.uk (A.S.); 2 Department of Pharmacology and Toxicology, School for Nutrition and Translational Research in Metabolism (NUTRIM), Maastricht University Medical Center, 6200 MD Maastricht, The Netherlands

**Keywords:** breath biopsy, volatile organic compounds (VOC), chronic liver diseases

## Abstract

The gold standard method for chronic liver diseases diagnosis and staging remains liver biopsy, despite the spread of less invasive surrogate modalities based on imaging and blood biomarkers. Still, more than 50% of chronic liver disease cases are detected at later stages when patients exhibit episodes of liver decompensation. Breath analysis represents an attractive means for the development of non-invasive tests for several pathologies, including chronic liver diseases. In this perspective review, we summarize the main findings of studies that compared the breath of patients with chronic liver diseases against that of control subjects and found candidate biomarkers for a potential breath test. Interestingly, identified compounds with best classification performance are of exogenous origin and used as flavoring agents in food. Therefore, random dietary exposure of the general population to these compounds prevents the establishment of threshold levels for the identification of disease subjects. To overcome this limitation, we propose the exogenous volatile organic compounds (EVOCs) probe approach, where one or multiple of these flavoring agent(s) are administered at a standard dose and liver dysfunction associated with chronic liver diseases is evaluated as a washout of ingested compound(s). We report preliminary results in healthy subjects in support of the potential of the EVOC Probe approach.

## 1. Introduction

Symptomatic manifestations of chronic liver diseases are often a consequence of fibrosis, which, independently from etiology, becomes relevant only at the advanced stages culminating in liver cirrhosis [1]. Therefore, asymptomatic progression is intrinsic of the pathophysiologic mechanism, making chronic liver diseases hard to detect at early stages [1,2]. The last three decades have seen a global ~30% death toll rise caused by liver cirrhosis, accompanied by a ~100% increase in prevalent cases of decompensated cirrhosis [3]. These numbers express the magnitude of the growing health burden represented by chronic liver diseases worldwide [1,3,4].

The main factors leading to cirrhosis are hepatitis B and C, alcohol-related liver disease and non-alcoholic steatohepatitis (NASH), with the latter expected to overtake hepatitis viruses in the near future [3]. These, together with less prevalent factors, promote disease progression to cirrhosis with shared mechanisms [5,6]. Signals from persistently injured hepatocytes, represented by reactive oxygen species (ROS) and lipid peroxidation products, promote hepatic stellate cell (HSC) transition from a quiescent to an activated, myofibroblast-like, state [7]. Perpetuation of the insult maintains and promotes survival, proliferation, and migration of activated HSC, which synthetize and deposit increasing amounts of collagen and fibronectin in the extracellular matrix, providing the main contribution to fibrotic tissue accumulation [5]. Fibrosis progression is accompanied by sinusoidal capillarization and formation of intrahepatic shunts, with progressive loss of functional parenchymal cells, until, histologically, the liver displays diffuse nodular regeneration surrounded by fibrotic septa at the cirrhotic stage [1]. 

Cirrhosis complications include esophageal varices and ascites as a consequence of portal hypertension; jaundice and hepatic encephalopathy resulting from hepatocellular insufficiency; and hepatocellular carcinoma (HCC) induced by sustained inflammatory damage and persistent hepatocytes necrosis and regeneration [8,9]. More than 50% of the subjects affected by chronic liver diseases receive their first diagnosis at the cirrhosis stage when they show one or more of these complications [1,2], indicating that more efficient screening strategies must be implemented to identify cases that remain undetected until overt symptoms occur [10]. 

Histopathologic analysis of liver biopsy represents the gold standard method for diagnosis and staging of chronic liver diseases [11], and the sole diagnostic method for NASH [12]. Nevertheless, given the invasiveness and risk of complications associated with the biopsy procedure [13], surrogate imaging modalities, and direct/indirect serum fibrosis tests are used singularly or in combination, with biopsy sporadically prescribed when non-invasive tests produce uncertain results [1,14]. Among the imaging modalities, transient elastography (FibroScan™, EchoSens™, Paris, France) is one of the most frequently applied for chronic liver diseases to measure fibrosis and changes in liver fat [15]. Given portability and device ease of use, transient elastography can be implemented in primary care, and is suitable for the screening of populations bearing chronic liver diseases risk factors [10]. However, a failure rate of ~16%, especially in patients with high body mass index (BMI), has been observed [15,16,17], together with a poor discrimination of pre-cirrhotic stages of fibrosis; hence, performance excels only in advanced fibrosis [18]. Multiparametric magnetic resonance imaging-based Liver Multi Scan (LMS) (Perspectum Diagnostics Ltd., Oxford, UK) is an emerging diagnostic tool that showed lower failure rate than FibroScan (4.3%), and offers the possibility to measure hepatic fibrosis, inflammation, iron, and as low as 1% changes in fat content, with similar FibroScan performances for fibrosis [17]. However, high costs, and massive hardware, demote LMS toward secondary care, making it inconvenient as a screening tool.

Fibrosis tests based on serum biomarkers are in general cost-effective compared to liver biopsy, and can be repeated multiple times, representing excellent alternatives to monitor treatment efficacy. Enhanced Liver Fibrosis (ELF) test (Siemens, 91052 Erlangen, Germany) combines 3 direct serum biomarkers of fibrosis (hyaluronic acid (HA) [19,20,21,22], tissue inhibitors of metalloproteinases (TIMP-1) [23,24,25,26,27,28,29], amino-terminal propeptide of procollagen type III (PIIINP) [30,31,32,33]), and shows good performance in significant and advance fibrosis [34]. FIB-4 combines indirect serum markers alanine aminotransferase (ALT) and aspartate aminotransferase (AST) together with platelets and age, and accurately predicts fibrosis in advanced stages [35]. Details about the full range of alternatives to liver biopsy, their applications and performance, can be found in references [1,15,36]. In summary, alternative methods avoid liver biopsy in the majority of the cases, at a cost of low sensitivity in early fibrosis stages [1].

Establishment of the exact liver disease etiology and stage is crucial to identify the most appropriate therapeutic strategy. Available treatments aim to alleviate complications and eradicate the underlying factor(s) of liver injury, in order to stop, delay, and when possible, reverse disease progression. In general, when chronic liver diseases are detected at the cirrhotic stage with complications, the hepatic damage is irreversible even after underlying factor eradication. Therefore, therapeutic interventions mainly focus on symptom management. Bleeding of esophageal varices is one of the deadly complications and requires fluid resuscitation, vasopressors, endoscopic band ligation, or injection sclerotherapy. Transjugular intrahepatic portosystemic shunt (TIPS) may increase survival rate in some cases [8]. Ascites are usually managed with diuretics and restriction of sodium intake [8], with severe cases (tense ascites) that require repeated paracentesis coupled with albumin replacement [37,38]. Treatment of hepatic encephalopathy aims to reduce blood levels of ammonia by administration of lactulose, which prevents its gastrointestinal adsorption, and rifaximin, which reduces production of ammonia by gut flora [8]. HCC, which often develops on a background of cirrhosis, requires semi-annual survey for early detection, and resection/ablation of the malignant nodule(s) at early stages [9].

Factor-specific treatments include viral suppression/eradication for viral hepatitis, alcohol abstinence for alcoholic liver disease, lifestyle changes consisting of weight loss and increased physical activity for NASH, and suppression of immune system for autoimmune hepatitis [8]. Interestingly, these treatments have the potential to block or reverse disease progression when administered at early stages, preserving liver function and preventing complications [1]. Therefore, more effective strategies for screening of at-risk populations must be implemented to detect chronic liver diseases at the pre-symptomatic stages.

## 2. Breath Analysis Is an Attractive Means for Chronic Liver Diseases Early Detection

Exhaled breath represents an excretion route for a subset of metabolites referred to as volatile organic compounds (VOCs). These VOCs originate from endogenous metabolism (e.g., acetone), gut microflora (e.g., indole), and environmental exposure (e.g., limonene). We refer to the latter as exogenous volatile organic compounds (EVOCs), which are often introduced in the body as xenobiotics through enteral or parenteral routes [39]. Boundaries between the origins of different VOCs are often not well defined.

Alterations of hepatic function, consequent to chronic liver diseases, change the rates of metabolic pathways involved in VOCs production or clearance, resulting in modifications of the abundance of these metabolites on breath. Thus, these VOCs represent potential biomarkers for a breath test. Evidence in support of this concept was already “perceived” by Hippocrates (460–370 B.C.), who described *fetor hepaticus*, the musty breath of subjects undergoing liver failure [40]. The last five decades have seen the evolution of more quantitative and accurate instruments that led to the discovery of a considerable number of breath compounds associated with several diseases [41,42,43], including Alzheimer’s [44,45], Parkinson’s [44], schizophrenia [46,47], multiple sclerosis [48,49], breast cancer [50,51], colorectal cancer [51,52,53], lung cancer [51,54,55,56,57,58,59,60], asthma [61,62,63,64,65], chronic obstructive pulmonary disease (COPD) [61,66,67,68,69,70,71], cystic fibrosis [72,73,74,75,76], and COVID-19 [77,78].

Chen et al. (1970) [79], Kaji et al. (1978) [80], and Tangerman et al. (1994) [81], compared the breath of patients with cirrhosis against that of healthy controls, by using gas chromatography (GC). They found significantly elevated levels of sulfuric compounds in the breath of patients with cirrhosis, with dimethyl sulfide identified as the main compound responsible for *fetor hepaticus*.

Friedman et al. (1994) [82] analyzed the breath of 24 patients with chronic liver disease of different etiology and stages, and 24 healthy controls, by using GC mass spectrometry (GC-MS). Consistent with previous studies [80,81], the level of another sulfuric compound, hydrogen sulphate, was elevated in the disease group. Additionally, 12 patients showed higher levels of limonene, 8 of which were affected by non-cholestatic liver diseases (i.e., hepatitis and cirrhosis). This novel compound of exogenous origin was associated with disease etiology, but also with the amount ingested through citrus products consumption, indicating that exposure represents an impactful confounding factor.

Sehnert et al. (2002) [83] analyzed the breath of 86 patients with chronic liver disease of different etiology and 109 subjects with normal liver function using GC. Stratification of patients based on disease etiology revealed that carbonyl sulfide was significantly increased either in patients with bile duct injury, or hepatocellular injury. While dimethyl sulfide and ethane were elevated only in the latter.

Van den Velde et al. (2008) [84] analyzed the breath of 52 patients with liver cirrhosis of different etiology and that of 50 healthy controls using GC-MS. A total of 12 discriminatory compounds were identified, of which the levels of dimethyl sulfide, acetone, 2-butanone, and 2-pentanone were increased, while indole and dimethyl selenide were decreased. Four of these (dimethyl sulfide, 2-pentanone, indole, and dimethyl selenide) were used to build a classification model with sensitivity and specificity of respectively 100% and 70% on a test set.

The same research group, in a follow up study (Dadamio et al. (2012) [85]), enrolled 35 cirrhotic patients and 49 healthy controls. GC-MS breath analysis identified 891 compounds, of which 67 were present in at least half of the samples. A total of 28 compounds showed significant differences between the study groups and were used to build classification models that returned a sensitivity and specificity of 83.3% and 100%, respectively, on a test set.

Pijls et al. (2016) [86] used a different study design, by enrolling 87 chronic liver disease pre-cirrhotic and 34 cirrhotic patients and 31 healthy controls. GC-MS breath analysis identified 3718 compounds, of which, a set of 23 and 19 differentiated controls from chronic liver disease and cirrhotic groups with a correct classification of 100% and 93.75%, respectively. An additional model based on a subset of 11 compounds discriminated pre-cirrhotic from cirrhotic patients with a sensitivity of 83%, specificity of 87% and area under the ROC curve (AUC) of 0.90 on a test set, indicating that hepatic metabolic alterations can be detected on breath at pre-cirrhotic stages.

Morisco et al. (2013) [87], enrolled 12 patients with cirrhosis and 14 healthy controls, and analyzed their breath using Proton Transfer Reaction Mass Spectrometry (PTR-MS), which compared to GC-MS used before, provides superior time resolution at the expense of compound identification based on mass only. A total of 285 mass peaks were identified, of which 51 showed significant differences between the two groups. A compound identified as a monoterpene showed the highest classification performance (AUC = 0.87). Stratification of patients based on disease severity, measured as Child-Pugh (CP) class, showed a trend of increase C8-Ketone in subjects with CP B and C. Consistently, the same compound showed a significant positive correlation with serum bilirubin.

Fernandez Del Rio et al. (2015) [88] applied an unprecedented mixed cross-sectional and longitudinal study design. For the cross-sectional component they enrolled 31 patients affected by advanced chronic liver disease and 30 controls. PTR-MS breath analysis identified five significantly different compounds, of which a combination of methanol, 2-pentanone, and limonene yielded the best classification between the groups with an AUC of 0.95. For the longitudinal component, they implemented a follow up analysis of breath collected at different time points from patients that underwent liver transplant. Of the five compounds initially identified, only limonene was still elevated a few days after transplant with levels that normalized in the following weeks. This study design provided evidence that a direct link exists between breath limonene levels and impaired liver function.

Sinha et al. (2020) [89] explored earlier stage liver disease by enrolling 15 patients with CP class A (non-alcoholic fatty liver disease (NAFLD)-cirrhosis), 14 with non-cirrhotic NAFLD, and 14 healthy controls and analyzed their breath using GC-MS. Limonene, dimethyl sulfide, and terpinene, normalized by BMI, gave the highest predictive accuracy between study groups. Cirrhotic patients were discriminated from pre-cirrhotic and healthy by limonene combined with dimethyl sulfide with AUCs of 0.91 and 0.98, respectively. Pre-cirrhotic were discriminated from healthy by terpinene with an AUC of 0.84. However, while the first two compounds were elevated in the disease groups, the latter was reduced.

Ferrandino et al. [90], in a targeted approach, investigated limonene in the breath of 44 patients with cirrhosis and 40 controls and found a discriminatory performance with an AUC of 0.78. Additionally, they investigated correlations between limonene and blood metrics of liver function/damage and found positive correlations with bilirubin and prothrombin time (PT-INR), and a negative correlation with albumin. However, no correlation was observed with ALT. Consistently, cirrhotic patients with CP B showed significantly higher levels of limonene compared to patients with CP A. Among the confounding factors, dietary exposure also impacts levels of limonene on breath [90] as described before [82]. In agreement with those data, the authors have previously reported that administration of limonene and other terpenes elevated their levels on breath for a short time in one healthy subject [39]. Limonene is a flavoring agent generally recognized as safe (GRAS), widely used in the food industry and abundant in citrus fruits. This compound has been identified in several previous studies as associated with chronic liver diseases and showed the highest classification performance compared to other compounds [82,85,86,87,88,89].

Hypothesized factors leading to elevated breath limonene in cirrhotic patients are, at the anatomical level, the impaired liver clearance due to portosystemic shunts and sinusoidal capillarization, associated with a reduced functional liver mass [90], and at the molecular level, downregulation of CYP2C9 and CYP2C19 activity [88], the main limonene metabolizing enzymes [91]. Thus, impaired conversion of limonene to hydrophilic bioproducts reduces its excretion through urine, leading to its accumulation in the adipose tissue [92], and increased excretion through the breath.

The studies summarized here, and reported in Table 1, support the following conclusions:

(1) Breath analysis could be used for chronic liver disease detection.
(2) Overall, a wide spectrum of chronic liver diseases and etiologies were explored.
(3) Limonene and other potential biomarkers with best classification performance and confirmed by multiple independent studies are of exogenous origin (Table 1, compounds in blue, and Table 2).
(4) Small sample size and lack of validation demote these compounds to the proof of principle stage.
(5) Additional studies with reliable power and implementing strategies to control the exposure confounder must be performed to transition these potential biomarkers from proof of principle to marketed breath tests.

## 3. Transitioning from Endogenous Biomarkers to Exogenous Reporters

Taking limonene as an example, available evidence about the association between breath limonene levels and liver dysfunction [82,85,86,87,88,89,90] supports availability of this compound as breath biomarker. However, inability to control exposure in the general population prevents the definition of on-breath thresholds for the identification of affected subjects. On one hand, disease subjects who conduct a lifestyle with low limonene exposure, (i.e., avoiding fruit juices, fruit, and beverages) may be misclassified as healthy, on the other hand, healthy subjects, who consumed a limonene-containing product shortly before testing may be misclassified as diseased. Whilst fasting subjects before testing controls dietary contribution, liver function may be expressed as limonene clearance, measured as a washout curve of administered compound in an experimental setting. Thus, subjects with compromised hepatic function display either higher baseline levels (before compound administration), or a slower washout.

A fundamental requirement for the design of an EVOC approach is the control of confounding factors [39]. For example, limonene is widely used as a fragrance in cleaning products and cosmetics. Therefore, environmental contamination may derive from the ambient air, perfumes, lipsticks, hand sanitizers, toothpaste, and mouthwash. All these confounders must be overseen in a study design by instructing the participants to avoid smoking, use mouthwash, brush teeth, and do not wear lipstick or perfumes before the test. Ambient contributions should always be controlled by collection of various blank samples. An accurate experimental design minimizes the impact of confounding factors, hence, false discovery rate.

Other approaches utilizing exogenous reporters showed potential for disease detection. For example, VOC-modified peptide substrates conjugated to polyethylene glycol (PEG) nanocarrier showed promising pre-clinical results for respiratory disease detection. During inflammation, neutrophils release neutrophil elastase (NE), a protease that cleaves the VOC reporter, which is detectable on breath within 10 min from nanocarrier delivery [98]. Exogenous reporters could also be detected in urine as shown by utilization of ezetimibe glucuronide (EZE-GLU), which is normally excreted through bile, which appears in the urine of NASH rats. The switch in excretion route takes place because of simultaneous downregulation of organic-anion-transporting polypeptides (OATP), which mediates uptake of the reporter in the hepatocytes; upregulation of multidrug resistance-associated protein 3 (MRP3), which transport EZE-GLU from hepatocytes into the sinusoid; and mis-localization of MRP2, which transport the compound in the bile ducts. Consequent increase of EZE-GLU retention in the systemic circulation, results in elevated urinary excretion [99].

## 4. Advantages of GRAS Compounds over ^13^C-Reporters Breath Tests, and Blood Tests

A dynamic approach to measure liver function with a breath test relies on the application of ^13^C-labeleld exogenous compounds (e.g., aminopyrine [100], caffeine [101], or methacetin [102]). In these molecules, ^12^C in a methyl functional group is replaced with the stable isotope ^13^C. Hepatic dealkylation of the labelled compound, mediated by CYP enzymes, produces ^13^C-CO_2_, which is exhaled in breath. Overall, 1% of exhaled CO_2_ contains ^13^C due to the natural abundance of the stable isotope. Therefore, the excess ^13^C-CO_2_ over baseline after compound administration is directly correlated with hepatic function.

The aminopyrine breath test was explored to evaluate liver function in patients with cirrhosis, to predict the outcome of acute liver failure, alcoholic hepatitis, and the survival in patients with cirrhosis. A total of 125 cirrhotic patients were followed for up to 48 months. During the follow up period 43 patients died (20 of liver failure, 15 of gastrointestinal bleeding, three of infections, two of hepatorenal syndrome, and three of diseases not related to cirrhosis). Cox’s multiple regression analysis identified aminopyrine breath test, Pugh-score, and etiology as best predictors of death for liver failure [103].

The caffeine breath test (CBT) showed potential for liver function evaluation in patients with chronic hepatitis B and C [104]. Most importantly, CBT showed a classification performance for advanced fibrosis (Metavir F ≥ 3), with an AUC of 0.91, in patients affected by chronic hepatitis B, and ability to identify responders to lamivudine treatment. CBT showed classification performance in NASH patients progressed to severe fibrosis (Brunt’s score ≥2) and cirrhosis with AUC of 0.788 [105] and 0.86 [106], respectively.

Among the different ^13^C labelled compounds, ^13^C-methacetin is the most promising in evaluating liver function [2]. In an initial application, the compound was administered orally. In hepatitis C subjects ^13^C-CO_2_ production was reduced only in patients with advanced fibrosis (Knodell score). However, the methacetin breath test (MBT) showed classification performance for non-symptomatic cirrhosis with a 75% sensitivity and 100% specificity [102]. Similar results were observed in NASH patients, both ^13^C-CO_2_ production at the 10 min peak, and cumulative 60 min after administration showed AUC > 0.9 for patients progressed to fibrosis stage ≥ 3 (Brunt’s score). The LiMAx test (maximum liver function capacity) (Humedics GmbH, Berlin, Germany) is the latest evolution of the ^13^C-methacetin breath test, in which the reporter compound is injected, and ^13^C-CO_2_ production is monitored in the following hour. The LiMAx test found its application in perioperative monitoring for hepatectomy, by predicting postoperative hepatic dysfunction and define the clinical decision tree for patient management [107].

The complexity of hepatic metabolic pathways does not allow to accurately evaluate liver function with one single compound. Although specific ^13^C-labelled compounds have been identified to test different metabolic processes as a proxy of liver function [108], all have ^13^C-CO_2_ as reporter, precluding the possibility of testing multiple pathways at once. This limitation does not apply to blood-based liver function tests, where measurement of multiple endogenous biomarkers from a single blood sample, and their combination in scoring systems is informative of the liver condition. However, alterations of these endogenous biomarkers often become evident only at advanced stages, when liver injury is well established, reducing chances for early detection [1].

The long list of chemicals approved as GRAS, and widely used as flavoring agents [109], includes compounds which are highly volatile and mainly metabolized by the liver (e.g., limonene, menthol, eucalyptol) [91,110,111]. Application of these compounds for a dynamic liver function breath test provides the manifold advantages of

(1) Assessing multiple metabolic routes at once, by administering combination of compounds metabolized by different pathways.
(2) Challenging the hepatic functional reserve, by administration of high, but still safe doses [110,112], which can reveal subtle metabolic alterations typical of early stages, yet undetectable by endogenous biomarkers.
(3) Increase technical sensitivity by administrating doses that provide a signal several folds over background levels.

For example, a phase 1 clinical trial showed that limonene is safe up to 15 g/day for one year [112], indicating that such a compound would be suitable also for multiple testing to follow disease progression or treatment efficacy.

## 5. Experimental Section: Preliminary Results from Healthy Subjects Support the Feasibility of the EVOC Probe Approach

To the awareness of the authors, only four studies published to date showed the profile of compounds on breath after oral administration [39,113,114,115]. In these studies, the participants ingested capsules containing one or more compounds, which were measured on breath post-administration. Administered compounds appeared on breath as expected, representing supporting evidence of the feasibility of a dynamic EVOC approach. However, in some instances, the breath kinetic was highly variable and hence could hardly be used for classification or staging of chronic liver disease patients. EVOC oral delivery through capsules may be disadvantageous because compound release requires coating dissolution, a process that may introduce variability, limit compound bioavailability, and extend time for testing [39].

To overcome these limitations, Owlstone Medical investigated breath profiles of several compounds administered in a liquid form. A proof of concept study was conducted, by enrolling three healthy individuals for each compound. Each individual performed the experiment in triplicate in non-consecutive days. Objectives of these preliminary experiments are:

1. Develop analytical capability for breath detection of these compounds.
2. Identify compounds that appear on breath after oral administration at the chosen doses.
3. Estimate time points and a time range for breath collection to observe a spike followed by a washout.

These requirements are essential to increase chances of success of the EVOC strategy in cross-sectional clinical trials, appropriately powered, enrolling chronic liver disease patients and controls.

**Figure 1:**
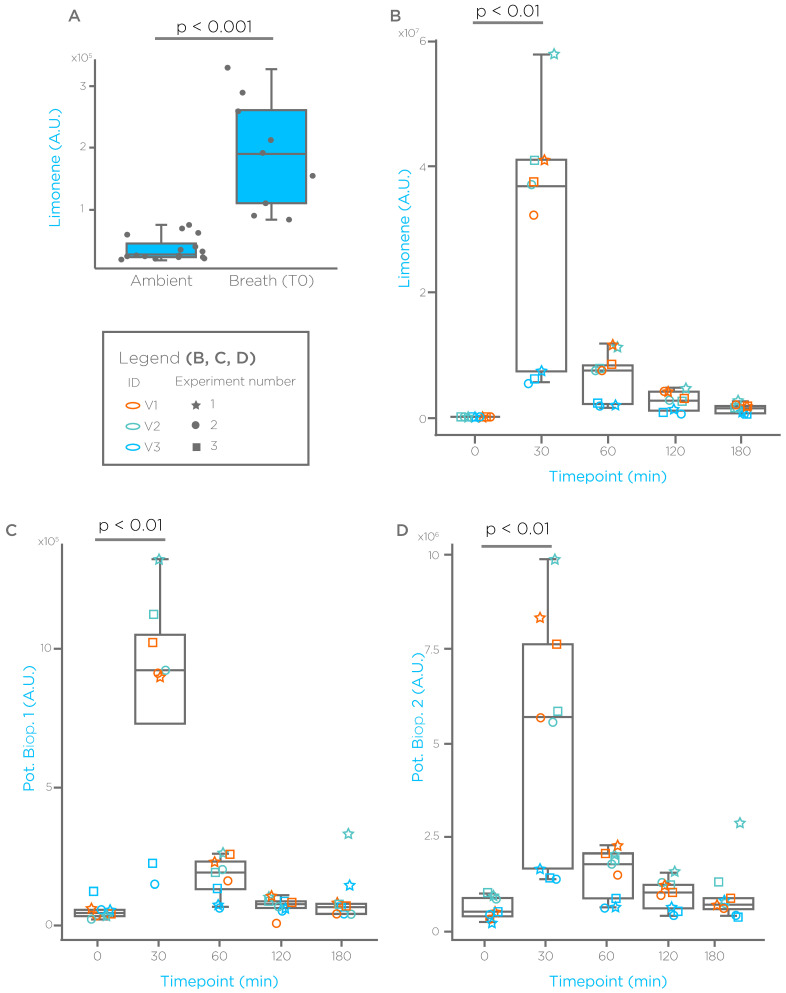
Limonene breath profile in healthy subjects before and after oral administration. (**A**) Breath limonene levels in 3 healthy subjects measured in triplicate after ≥10 h fasting and compared to ambient. (**B**) Breath limonene levels measured before and after oral administration at the indicated time points. Limonene ingestion induces a spike in breath after 30 min followed by a progressive reduction toward baseline levels. (**C**,**D**) Spectral features showing a limonene washout-like time course, which could be potential bioproducts (Pot. Biop.). A.U. = arbitrary units.

As shown in Figure 1A, healthy subjects, after an overnight fasting (>10 h) showed breath limonene levels significantly higher than ambient. After administration (Figure 1B), limonene levels showed a significant spike at 30 min, with a reduction after 1 h, with levels that tended toward baseline after 2 and 3 h. Interestingly, exploration of chromatograms using Breath Biopsy^®^ OMNI identified spectral features showing a similar profile to that of limonene (Figure 1C,D), which may be potential volatile limonene bioproducts.

**Figure 2:**
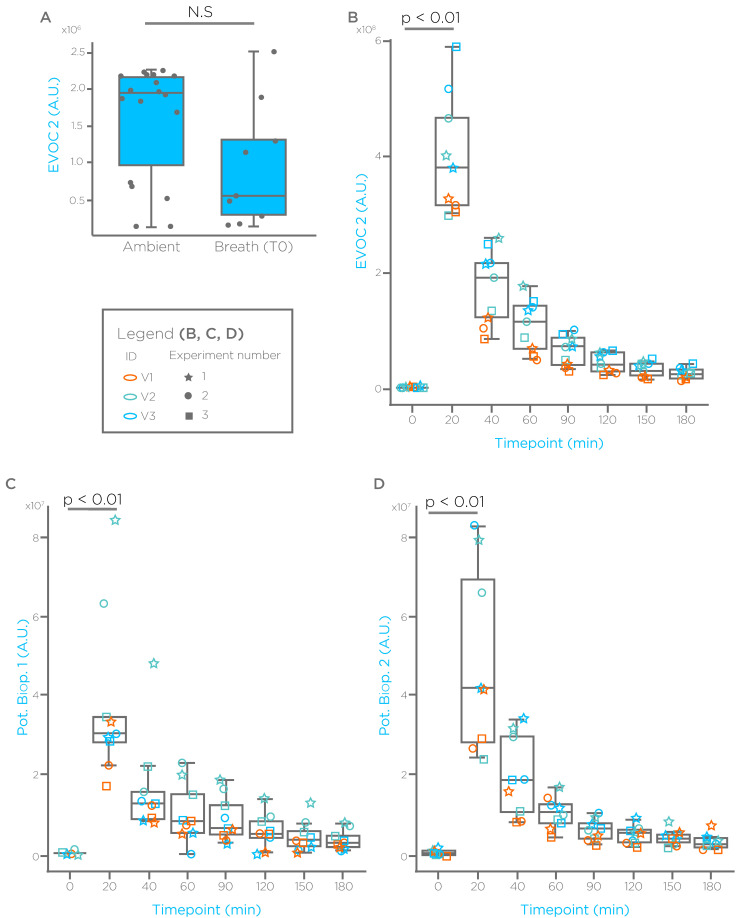
EVOC 2 breath profile. (**A**) Levels of EVOC 2 in ambient and breath from 3 healthy subjects fasted for ≥10 h. (**B**) EVOC 2 profile on breath before and after administration at the indicated time points. (**C**,**D**) Spectral features representing potential EVOC 2 bioproducts (Pot. Biop.). A.U. = arbitrary units.

**Figure 3:**
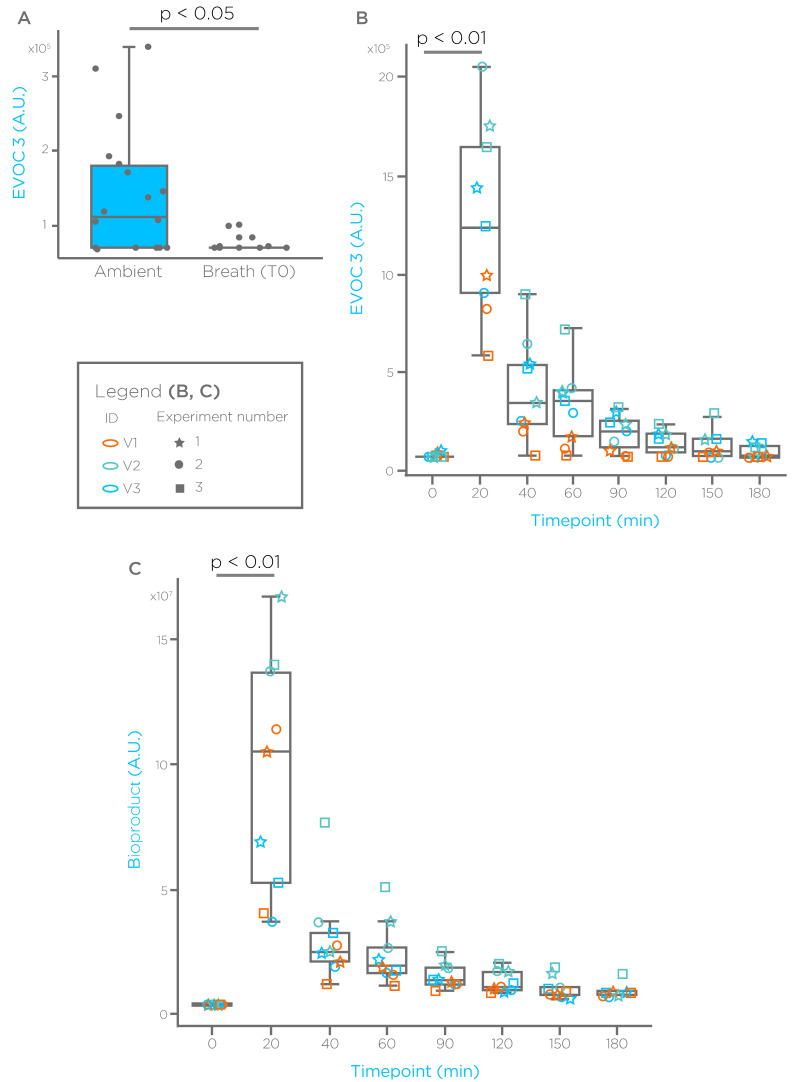
EVOC 3 breath profile. (**A**) EVOC 3 breath levels in ambient and breath of fasted subjects. (**B**) EVOC 3 breath profile before and after administration. (**C**) Breath profile of a known EVOC 3 bioproducts (Pot. Biop.). A.U. = arbitrary units.

With a similar approach we have tested additional, non-terpene GRAS compounds that were found elevated in the breath of cirrhotic subjects. We report results for two of these compounds in Figure 2 and Figure 3, where we refer to them as EVOC 2 and EVOC 3. EVOC 2 showed no significant differences between ambient, and breath taken from fasted, healthy subjects (Figure 2A). As observed already for limonene, EVOC 2 administration generates a spike on breath after 20 min, with levels that reduce in the following hours (Figure 2B). Exploration of chromatograms revealed additional spectral features with a similar EVOC 2 profile, which may represent potential bioproducts. Interestingly, breath baseline levels of EVOC 3 after an overnight fasting were significantly lower than ambient (Figure 3A). However, also for this compound we observed a significant spike 20 min after administration, and a washout in the following time points (Figure 3B). We have also monitored a known bioproduct for EVOC 3, which showed a washout-like shape as well (Figure 3C).

The results obtained by these preliminary, pilot experiments indicate that the tested compounds are suitable for further investigation in cross-sectional clinical trials at the tested doses and time points, as we observed that

(1) All the compounds showed baseline breath levels close or similar to ambient after an overnight fasting, suggesting that implementation of this procedure, reduces the confounder represented by random dietary exposure.
(2) Oral administration of the compounds via a liquid formulation induced a spike on breath in all the subjects and in all the experiments, overcoming unreliable appearance on breath we experienced using capsules (data not shown), providing the benefit of detecting the compounds more easily, and avoiding the confounder represented by ambient contamination.
(3) The reduction observed after 40–60 min suggest that the absorption phase is mainly completed within the first hour.

Hypothesizing that the levels of compounds on breath after one hour are mainly dependent on the functional capacity of the liver, subjects affected by chronic liver diseases with reduced liver function, are expected to show a delayed washout with higher levels of compounds on breath at later time points; this difference enables classification. Additionally, identification and characterization of the identified potential bioproducts is essential to evaluate their utility for a breath test. Overall, these results add to the feasibility of the EVOC strategy. However, additional experiments on larger cohorts, and involving subjects with chronic liver diseases, are essential to assess validity of this strategy for a breath test.

## 6. Discussion

Given the impact of chronic liver diseases on the hepatic metabolic capacity [3] and their inefficient detection at the asymptomatic stages [10], additional diagnostic methods must be developed to fill the gap of current strategies. Breath analysis has the potential to fill this gap and be established for several applications:

**As a screening test:** Implemented in primary care and administered at regular intervals to detect potential liver dysfunction in the general population. In a dynamic configuration, reporter compound(s) are measured in breath before administration, when levels in fasted subjects are expected to be close to ambient contamination, and at a specific time point (reasonable for a point of care or home-based testing), when levels are expected to be below a certain threshold. Crucial requirement for this application is to establish this threshold in the healthy population. The main competition and barrier to the market is blood-based liver function tests, which, despite low sensitivity and specificity [116], is routinely prescribed for its ease of use and interpretation, and cost-effectiveness. Although a breath test could outperform the liver function test in terms of diagnostic performance, especially in earlier stages, development phases must take into account final costs and ease of use to ensure success for this application. Hence, a key advantage of a breath test is the possibility to obtain real time results [117], eliminating the need of a second visit.

**As a diagnostic test:** Used as a supportive diagnostic (or recruitment enrichment) tool, administered to populations bearing risk factors to identify potentially affected subjects who are eligible for further confirmation of diagnosis with imaging modalities and/or biopsy. A crucial factor for this application is the development of a cost-effective breath test with high sensitivity and specificity, in order to efficiently select subjects undergoing further investigation, such as costs of a breath test implementation in clinical practice offset savings generated by a reduced number of expensive confirmatory tests.

**As a prognostic test:** Acting as a proxy for the occurrence of future adverse liver events enabling identification of progression of chronic liver diseases to more advanced stages, or to monitor disease regression in approved or experimental therapeutic interventions. Essential requirement for this application is the capability of the breath test to detect subtle changes in chronic liver disease stages. This sensitivity can be achieved by a combination of several compounds targeting different metabolic pathways that show alterations across the severity spectrum [118]. Experimental drug treatments, especially for NASH, suffer from lack of non-invasive and reliable tests for endpoint efficacy evaluation. A reliable breath test could replace liver biopsy, currently used for this purpose, overcoming its limitations [12].

For any of these applications, adoption of a breath test in clinical practice requires robust evidence supporting clinical validation, and performances that are similar or improved compared to existing modalities. Other factors that impact clinical adoption are Food and Drug Administration (FDA) approval, insurance coverage, inclusion in guidelines, and clinical utility.

Breath analysis has been applied in clinical practice for breath tests such as hydrogen-methane test (for gastrointestinal diseases), ^13^C urea test (for *Helicobacter pylori*), the Heartsbreath test (for heart transplant rejection), breath carbon monoxide test (for neonatal jaundice), and exhaled nitric oxide test (for asthma) [119]. This perspective review aims to gather evidence of the feasibility of a breath test for chronic liver diseases. Available data support the claim that compounds measured on breath can differentiate healthy subjects from patients affected by chronic liver diseases. Not surprisingly, compounds with the highest performance are of exogenous origin, given the xenobiotic detoxification function of the liver. Thus, transitioning from a steady state to a dynamic set-up (the EVOC Probe approach) is essential to overcome the confounder represented by random exposure. The path from proof of principle to an approved breath test still entails key steps and requires identification of compounds that meet key requirements for this application. These compounds must benefit from a safety profile for repeated administration at higher doses compared to random dietary exposure. Gastrointestinal adsorption must be fast in order to deliver the whole dose in a short period of time. This contributes to overload the hepatic pathways and shorten the time required for the test. Here we provided evidence that shifting from capsules to a liquid form fulfil these requirements. Additionally, pharmacokinetic, hepatic extraction capacity, clearance, and biotransformation of reporter compounds must be fully characterized for test optimization.

In conclusion, transitioning the EVOC strategy from proof of principle to reality represents a challenging task. However, the resulting benefit for chronic liver diseases early detection will change the diagnostic pathway, improving the patient’s quality of life.

## 7. Materials and Methods

### 7.1. Participants and Protocol

All participants provided written informed consent. Three subjects for each compound, older than 18 years old, non-smokers over the last 6 months, not on prescription or over-the-counter drugs, and weighing more than 60 kg were instructed before the experiment to fast for ≥10 h, to not consume alcohol the previous day, and to not brush teeth or use mouthwash in the previous 2 h. Participants provided a first breath sample followed by one compound administration in a liquid form by using an oral fluid medicine syringe (BD Discardit II 309050 BD), followed by 200 mL of water to wash potential residual compound from the mouth. Then post-administration breath samples were collected at the scheduled time points. Experiments were repeated 3 times on non-consecutive days with the same subjects for each compound.

### 7.2. Breath Biopsy Collection

Breath collection was performed in a single room at Owlstone Medical (Cambridge, UK) for all the subjects involved in the study. Breath samples were collected by adsorption onto C2-CXXX-5149 Bio-monitoring-inert-coated tubes with Tenax TA/carbograph 5TD adsorbent material (Markes International, Llantrisant, UK) through the ReCIVA^®^ Breath Sampler (Owlstone Medical, Cambridge, UK) [90]. Before sampling, the tubes were conditioned in a TC-20 (Markes International) by a N_2_ flow at 20 psi and 320 °C for 2 h. Before sampling, the system was calibrated and adjusted based on the breathing pattern of the subject. Approximately 500 mL of breath was sampled per tube at 225 mL/min. Ambient contamination was minimized by using the CASPER^®^ Portable Air Supply (Owlstone Medical, Cambridge, UK) [90]. Additional ambient blanks were collected for each session, inside the room where the breath collection was performed, to track any environment contamination. Ambient collection was performed with a similar procedure used for breath sampling except that room air was directed in the sorbent tubes bypassing the CASPER air supply.

The tubes were purged using a TD-100 (Markes International Ltd. Llantrisant, UK) and stored at a temperature of 4–8 °C for no more than 4 weeks before analysis.

### 7.3. Analytical Methodology

Samples were analyzed using a Q Exactive mass spectrometer coupled with a Trace 1310 gas chromatograph (Thermo Fisher Scientific Inc. Waltham, MA, USA) and a TD100-xr thermal desorption system (Markes International Ltd., Llantrisant, UK). For the analysis of the samples two methods were used.

First method (Limonene): Samples were first pre-purged for 1 min. at 50 mL/min, then desorbed at 210 °C for 10 min at the same flow and focused onto a cold trap (U-T12ME-2S, Material Emissions, C4-C32 Markes International Ltd., Llantrisant, UK) at 20 °C. Analytes were then desorbed at 300 °C for 3 min into the GC column (TG-624SilMS 30 m × 0.25 mm × 1.4 µm, Thermo Fisher Scientific, Waltham, MA, USA) at 2 mL/min helium flow with a split ratio on the injection of 10:1. The GC temperature program was as follows: 40 °C held for 1 min, increasing to 270 °C at a rate of 10 °C/min, then to 300 °C with a rate of 30 °C/min and held 5 min. The ion mass detection was performed using an electron ionization (EI) mode with a mass scan range from *m/z* 30 to 450 with resolution of 60,000. EI voltage was set at 70 eV. The ion source and transfer line temperatures were 230 and 250 °C, respectively.

Second method (EVOCs 2 and 3): Samples were first pre-purged for 1 min at 50 mL/min, then desorbed at 250 °C for 10 min at the same flow and focused onto a cold trap (U-T12ME-2S, Material Emissions, C4-C32 Markes International Ltd. Llantrisant, UK) at 20 °C. Analytes were then desorbed at 250 °C for 4 min into the GC column (Stabilwax-DA 30 m × 0.25 mm × 0.25 µm, Restek Corporation, Bellefonte, PA, USA) at 2 mL/min helium flow with a split ratio on the injection of 3.5:1. The GC temperature program was as follows: 40 °C held for 2 min, increasing to 250 °C at a rate of 15 °C/min, and held for 5 min. The ion mass detection was performed using EI with a mass scan range from *m/z* 30 to *m/z* 450 with resolution of 60,000. EI voltage was set at 70 eV. The ion source and transfer line temperatures were 270 and 250 °C, respectively.

Together with breath samples, analytical grade standard solutions of the compounds under investigation were analyzed at different concentrations for the mass spectral identification and correction for fluctuations of instrument sensitivity.

## 8. Data Analysis

Peak area for each compound and its potential bioproducts was normalized to account for variations of instrument sensitivity as follow: For each sequence, the slope of standard curves was calculated using analytical grade standard solutions at different concentrations of the compounds under investigation, a slope ratio for each sequence and for each compound was calculated. Area of the compounds and their potential bioproducts were normalized for the slope ratio of the respective standard. The resulting value was expressed as arbitrary units (A.U.)

For the targeted analysis compounds identification and data investigation were performed using the Chromeleon software (Chromeleon 7.2.10, Thermo Fisher Scientific, Waltham, MA, USA).

For the untargeted analysis, the raw data were processed using Compound Discoverer 3.2.3.2.0.421 (Thermo Fisher Scientific, Waltham, MA, USA), to obtain a matrix of features present across all samples. The peak detection parameters were selected using 5 ppm accurate mass tolerance, 3:1 mass spectral signal to noise threshold, 1,000,000 of total ion chromatogram threshold and 97% of ion overlap window. For the group compound settings, the retention time tolerance was set at ± 2 s, with a dot product threshold of 500 and composition threshold of 15%.

A Mann–Whitney U test was used to test statistical differences between breath and ambient. Potential bioproducts were selected by using a paired Mann-Whitney test that compares the baseline breath levels (before compound administration), and the levels of the first time point post administration. Data were analyzed using R-studio (R Core Team (2020). R: A language and environment for statistical computing. R Foundation for Statistical Computing, Vienna, Austria. URL https://www.R-project.org/, accessed on 4 September 2021). Plots were generated using ggplot2 (H. Wickham. ggplot2: Elegant Graphics for Data Analysis. Springer-Verlag, New York, NY, USA, 2016).

## Figures and Tables

**Table 1:** Summary of studies that compared the breath of subjects with chronic liver diseases, against controls. Compounds in bold and blue are of exogenous origin. Arrows indicate if a compound is elevated or reduced in the breath of disease subjects compared to controls. CLD = chronic liver diseases.

Author/Year	Study Design	Analytical Method	Discriminant VOCs	Calssification Performance
Friedman et al. (1994) [82]	24 CLD 24 Controls	GC-MS	Hydrogen-sulphide ↑ Limonene ↑	n/a
Sehnert et al. (2002) [83]	86 CLD 109 Controls	GC	Carbonyl sulphide ↑	n/a
Van den Velde et al. (2008) [84]	52 CLD 50 Controls	GC-MS	Acetone ↑ Dimethyl-sulphide ↑ 2-butanone ↑ 2-pentanone ↑ Indole ↓ Dimethyl-selenide ↓	100% sensitivity 70% specificity
Dadamio et al. (2012) [85]	35 CLD 49 Controls	GC-MS	Dimethyl-sulphide ↑ Acetone ↑ 2-butanone ↑ 2-pentanone ↑ Indole ↓ Phenol ↓ Dimethyl-selenide ↓ Isoprene ↑ Ethane ↑ Pentane ↑	83% sensitivity 100% specificity
Pijls et al. (2016) [86]	34 cirrhotic 87 non-cirrhotic 31 controls	GC-MS	Dimethyl-sulphide ↑ Terpene (limonene) ↑ 2-methyl-butanal ↓ Propanoic acid ↑ Octane ↑ Terpenoid ↑ 3-carene ↑ 1-hexadecanol ↓ C_16_H_34_ ↓	83% sensitivity 87% specificity
Morisco et al. (2013) [87]	12 CLD 14 Controls	PTR-MS	Heptadienol ↑ Methanol ↑ 2-butanone ↑ 3-pentone ↑ 2-octanone ↑ 2-nonanone ↑ Monoterpene ↑ P-cymene ↑	83% sensitivity 86% specificity
Fernandez Del Rio et al. (2015) [88]	31 CLD 30 Controls	PTR-MS	Methanol ↑ 2-butanone ↑ Carbon-sulphide ↑ 2-pentanone ↑ Limonene ↑	97% sensitivity 70% specificity
Sinha et al. (2020) [89]	15 chirrosis NAFLD 14 non-cirrhosis NAFLD 14 Controls	GC-MS	Styrene Acetone Isoprene DMS D-limonene Acetophenone Terpinene	Cirrhotic vs. Control: AUCs = 0.98 Cirrhotic vs. Non-cirrhotic: AUC = 0.91 Non-Cirrhotic vs. Control: AUC = 0.84
Ferrandino et al. [90]	44 cirrhosis 42 controls	GC-MS	Limonene	AUC = 0.78

**Table 2:** Dietary sources and metabolic pathways involved in the biotransformation of exogenous VOCs found discriminant for chronic liver diseases. ? indicated the pathway has not been fully elucidated.

Discriminant VOC	Dietary Sources	Metabolizing Pathway(s)	Main Bioproduct(s)
Limonene	Fruit, fruit juices, citrus products	CYP2C9/CYP2C19 [91]	perillyl alcohol [91] trans carveol
2-butanone	Ripe fruit	Cytochrome P450 system [93]	3-hydroxy-2-butanone and 2,3-butanediol [93]
2-pentanone	Fruit, cheese, whiskey	Alcohol dehydrogenases (ADH) [94] ?	2-pentanol [94] ?
2-methyl-butanal	Crystal malts, baked potatoes, whole milk powder	Aldehyde dehydrogenases (ALDH) [95]	2-methyl-butanol [95]
Propanoic acid	Butter, cheese	Propionyl-CoA carboxylase (PCC) [96]	D-methylmalonyl-CoA [96]
1-hexadecanol	palm or coconut oil	Cytochrome P450 system [97]	Palmitic acid [97]
Acetophenone	Fruit	Glucuronosyltransferase (UGT) hippuric acid metabolism [97]	1-phenylethanol-glucoronide hippuric acid [97]
